# A new strategy for osteoarthritis therapy: Inhibition of glycolysis

**DOI:** 10.3389/fphar.2022.1057229

**Published:** 2022-11-10

**Authors:** Chunmei Tan, Lanqing Li, Juanjuan Han, Kang Xu, Xianqiong Liu

**Affiliations:** Hubei Engineering Technology Research Center of Chinese Materia Medica Processing, College of Pharmacy, Hubei University of Chinese Medicine, Wuhan, China

**Keywords:** osteoarthritis, glycolysis, enzymes, natural products, metabolic

## Abstract

Osteoarthritis (OA) is a common degenerative disease of the joints. It is primarily caused by age, obesity, mechanical damage, genetics, and other factors, leading to cartilage degradation, synovial inflammation, and subchondral sclerosis with osteophyte formation. Many recent studies have reported that glycolysis disorders are related lead to OA. There is a close relationship between glycolysis and OA. Because of their hypoxic environment, chondrocytes are highly dependent on glycolysis, their primary energy source for chondrocytes. Glycolysis plays a vital role in OA development. In this paper, we comprehensively summarized the abnormal expression of related glycolytic enzymes in OA, including Hexokinase 2 (HK2), Pyruvate kinase 2 (PKM2), Phosphofructokinase-2/fructose-2, 6-Bisphosphatase 3 (PFKFB3), lactate dehydrogenase A (LDHA), and discussed the potential application of glycolysis in treating OA. Finally, the natural products that can regulate the glycolytic pathway were summarized. Targeting glucose transporters and rate-limiting enzymes to glycolysis may play an essential role in treating OA.

## Introduction

Osteoarthritis (OA) is a degenerative joint disease characterized by articular cartilage destruction and synovial inflammation (Shen et al., 2012). OA mostly develops in the knee, followed by the hand and hip joints (Xu et al., 2021a). OA affects the entire joint structure, including articular cartilage, subchondral bone, meniscus, synovium, ligament, and sub-patellar fat pad (Pereira et al., 2015). It is characterized by pathological changes in joint structure, including cartilage degradation, synovial inflammation, and subchondral sclerosis with osteophyte formation (Zheng et al., 2021). It is featured by joint pain and stiffness, which reduces the quality of life (Yunus et al., 2020). Recent reports have shown that about 240 million people suffer from OA worldwide, and the incidence of OA among women is about 1.5 times that of men (Neogi & Zhang, 2013). Age, obesity, mechanical injury, and heredity are essential factors promoting OA development. However, the pathogenesis of OA remains unclear (Johnson and Hunter, 2014). Studies have shown that the incidence of the disease is high in the age group of 50–54 years, and the prevalence of men and women over 60 years old is 10% and 18%, respectively (Neogi and Zhang, 2013). Obesity can lead to joint overload and promote OA development. In addition, mechanical damage to articular cartilage and subchondral bone promotes OA development (Balahura et al., 2020; Gao et al., 2021). Part of OA is attributable to inheritance, with hereditary OA due to mutations in collagen type II, IX, or XI (Kannu et al., 2010).

Modern treatment of OA primarily includes surgical and non-surgical treatments (Tejero et al., 2021). Surgical treatments include peri-knee osteotomy and joint replacement (Madry, 2022). It can relieve pain, correct joint deformity, and restore joint function. However, problems exist, including high operation costs, high risk, and many other complications such as joint loss and infection. Non-surgical treatment includes standard treatment and medication, including rational exercise, weight control, and physical therapy. Drug therapy includes oral and topical administration and intra-articular injection (Rodriguez-Merchan, 2018). Oral non-steroidal anti-inflammatory drugs (NSAIDs) and opioids can relieve OA pain effectively (Paterson and Gates, 2019). However, long-term use could cause stomach and cardiovascular problems and kidney and liver damage. Topical application of NSAIDs or Chinese herbal medicines can provide anti-inflammatory and pain-relieving effects, although there are occasional adverse reactions such as local skin rash, burning sensation and itching. For patients with poor response to oral and topical drug treatment, intra-articular irrigation and injection therapy can be used, and the drug can act directly on the lesion site to relieve pain and improve joint function (Hameed and Ihm, 2012). However, repeated treatment can cause damage to the tendon and ligament of the articular cartilage. Therefore, developing alternative drugs with good efficacy and fewer side effects for treating OA is vital.

Modern studies have shown that metabolic disorders contribute to OA development (Sellam and Berenbaum, 2013). Regulating metabolism plays a key role in the treatment of OA (Mobasheri et al., 2017). There is a close relationship between OA and lipid metabolism (Cao et al., 2022), and massive lipid deposition is observed in osteoarthritic chondrocytes (Lippiello et al., 1991; Gkretsi et al., 2011). Some adipokines can directly affect joint health and regulate inflammation to promote OA progression (Wang & He, 2018). Amino acid metabolism is thought to be involved in the pathogenesis of OA (Li et al., 2016b), and a variety of amino acids are abnormally expressed in OA chondrocytes (Mickiewicz et al., 2015; Zheng et al., 2017). Unlike most tissues, articular cartilage has no blood vessels, nerves, or lymphatic vessels and is composed mainly of extracellular matrix (ECM) and chondrocytes (Wang et al., 2020b). ECM mainly consists of water, collagen, and proteoglycans (Sophia Fox et al., 2009). Studies have shown that the primary energy source of chondrocytes is glycolysis (Lane et al., 2015), and disorders of glycolytic metabolism can lead to chondrocyte hypertrophy and extracellular matrix degradation, promoting OA development (Kudelko et al., 2016). Generally, in healthy joints, chondrocytes are in metabolic balance (Wang et al., 2020b). In OA joints or inflammatory environments, chondrocytes undergo metabolic reprogramming, enhancing the glycolytic pathway. Glucose is transported into chondrocytes by the glucose transporter 1 (GLUT1), the first rate-limiting step in glycolysis (Peansukmanee et al., 2009). Glycolysis involves a variety of enzymes and continuous enzymatic reactions. HK2, PKM2, PFKFB3, and LDHA are abnormally expressed in OA chondrocytes (Errea et al., 2016; Qu et al., 2016; Wang et al., 2020c). These enzymes may serve as potential targets for OA treatment. Lactate, a metabolite of glycolysis, can block metabolic reprogramming and pro-inflammatory signaling pathways that play a pro-inflammatory role (Wang et al., 2020a). In addition, histone modifications reduce M1 macrophage activation and promote M2 macrophage polarization (Zhang et al., 2019; Ivashkiv, 2020). It regulates inflammation through a variety of mechanisms. Accumulating evidence suggests that the metabolic shift to glycolysis enables cells to gain energy and is vital for activating immune responses and inflammatory pathways in OA (Loftus and Finlay, 2016). Therefore, targeting the glycolytic switch, especially the glucose transporters and rate-limiting enzymes involved in this metabolic reprogramming, could be a massive breakthrough in OA therapy. Studies have found that natural products could regulate glycolysis (Duya et al., 2022; Kooshki et al., 2022), interfere with chondrocyte metabolic reprogramming, improve chondrocyte viability, and inhibit OA development. Moreover, natural products have the advantages of low toxicity, low cost and multiple targets (Gao et al., 2021).

This paper discussed the rate-limiting enzymes and metabolites involved in chondrocyte glycolysis that may have significant breakthroughs in OA treatment. In addition, we systematically summarized the natural products that can regulate glycolysis. The aim was to develop alternative drugs to effectively treat OA with fewer side effects.

## Glycolytic metabolism

During glycolysis, glucose molecules are taken up through the GLUT1. One glucose molecule is converted into two pyruvate molecules. These two pyruvate molecules relate to other metabolic pathways. They can enter mitochondria under aerobic conditions, where they are converted into acetyl CoA by pyruvate dehydrogenase complex and then completely metabolized into CO_2_ by the TCA cycle, generating NADH and reducing FADH2 to oxidative phosphorylation (OXPHOS) (Li et al., 2022b). Under anaerobic conditions, pyruvate is converted to lactate in the cytoplasm to regenerate NAD+, called anaerobic glycolysis (Zheng et al., 2021). Lactate formation from pyruvate under normoxic conditions is known as aerobic glycolysis or the Warburg phenomenon (Li et al., 2022a). High-throughput glycolysis occurs primarily in the cytoplasm, and lactate production from pyruvate is driven by lactate dehydrogenase (LDH) (Sotelo-Hitschfeld et al., 2012). LDH functions as a tetramer of LDHA and LDHB subunits. LDHB facilitates the conversion of lactate to pyruvate. LDHA is a rate-limiting enzyme with a high affinity for pyruvate and catalyzes lactate production from pyruvate (Feng et al., 2018). Since ATP production by glycolysis is much less efficient than OXPHOS, ATP production and lactate accumulation are reduced when OXPHOS is inhibited (Pucino et al., 2017).

## Osteoarthritis and glycolytic metabolic disorders

OA is the most common joint disease. OA has long been considered a degenerative disease caused by daily wear and tear of joints, primarily due to mechanical factors (Ahmad et al., 2020). However, recent evidence has shown that OA is a multifactorial disease. The development of OA involves mechanical, genetic, metabolic and inflammatory processes (Chen et al., 2017). During OA, inflammation is primarily driven by damage-associated molecular patterns (DAMPs). DAMPs are released in extracellular mediators after cell stress or injury and interact with pathogen-recognition receptors (PRRs), including Toll-like receptors (TLRs) (Ahmad et al., 2020; Lambert et al., 2020) and the receptor for advanced glycation end products (RAGE) to stimulate macrophage and chondrocyte activation (Xie et al., 2013; Lambert et al., 2020; Suzuki et al., 2022). Moreover, they produce inflammatory cytokines and chemokines (Rigoglou and Papavassiliou, 2013; Choi et al., 2019; Rai et al., 2022). The generated inflammatory mediators stimulate cartilage-degrading enzyme production and the recruitment of inflammatory cells (Berenbaum and Walker, 2020; Molnar et al., 2021), inhibit the synthesis of proteoglycan and collagen, regulate chondrocyte apoptosis, and promote joint degradation (Ridnour et al., 2007; Abramson, 2008). The degree of inflammation in OA is relatively low, but inflammation is considered an essential component of OA pathology and plays the driving role in joint destruction and OA progression (Siebuhr et al., 2016). OA is now regarded as a metabolically related disease. The establishment and OA development are associated with inflammation and metabolic changes (Berenbaum et al., 2017). Metabolism plays a vital role in OA development.

In OA, low-grade inflammation causes chondrocytes to be hypoxic, and energy metabolism changes from a resting regulated state to a metabolically active state (Kim et al., 2015). At this time, glycolysis levels increase to meet the energy requirements of chondrocytes (Maneiro et al., 2003). The primary energy source of OA chondrocytes is glycolysis (Li et al., 2020c). Articular cartilage, unlike most tissues, has no blood, nerve, or lymphatic vessels. The nutrients are provided by synovial fluid in the joints. Compared to plasma, oxygen and glucose are less available (Mobasheri et al., 2008). Its environment is relatively anoxic, and the energy produced by oxidative phosphorylation (OXPHOS) is low (Zhai, 2019). Glycolysis is a rapid process of ATP production (Vazquez et al., 2010). Therefore, chondrocytes are heavily dependent on glycolysis, and disorders of glycolytic metabolism can lead to chondrocyte hypertrophy and ECM degradation. This cellular metabolism is essential for energy balance and may also be critical for cell function and signaling changes (Kim, 2018). Increasing evidence indicates that metabolic disorders are the cause of OA (Sellam and Berenbaum, 2013). Therefore, regulating cell metabolism is crucial to prevent and treating OA.

## Targeted regulation of glycolytic metabolism to relieve osteoarthritis

**FIGURE 1:**
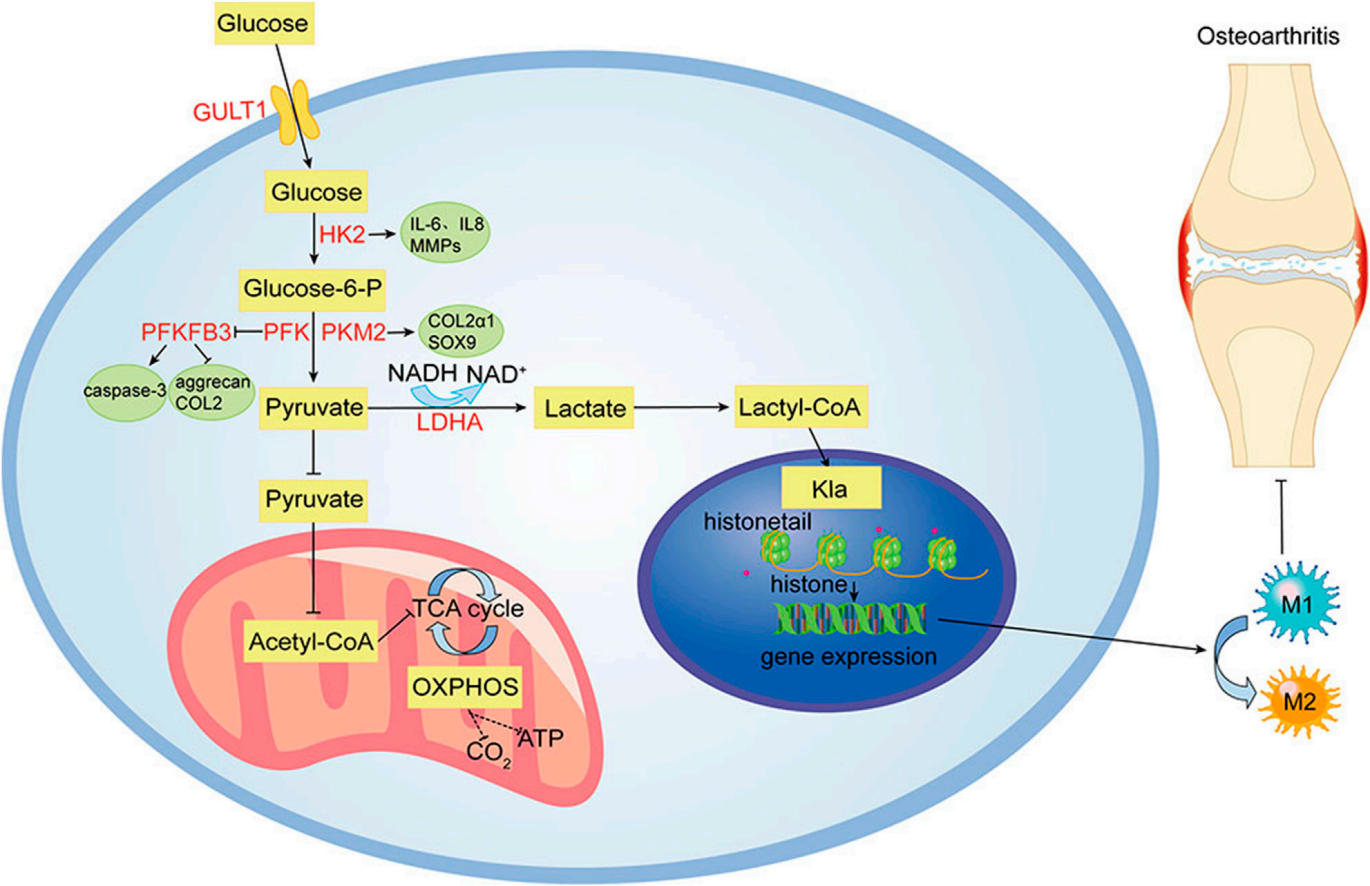
Glycolytic metabolism pathways of OA. Under hypoxic and inflammatory conditions in OA joints, cellular glycolysis is enhanced for rapid energy production. Many enzymes involved in glycolysis, including HK2, PKM2, PFKFB3, and LDHA, are abnormally expressed in OA chondrocytes. Lactate, a metabolite of glycolysis, reduces M1 macrophage activation and promotes M2 macrophage polarization by modifying histones.

Despite ongoing efforts to understand the pathogenesis and treatment of OA, to date, there is no cure for OA because cartilage is difficult to recover after being damaged (Xu et al., 2021b). The main objectives of modern OA treatment are to reduce pain, improve or maintain joint function, increase joint strength, and prevent further OA development (Xu et al., 2019). Clinically, approaches to treating OA have limitations in efficacy and long-term safety (Messina et al., 2019). Therefore, there is an urgent need to develop alternative drugs with few side effects that can effectively stop the progression of OA. Glycolysis plays a crucial role in OA. Its process includes a variety of enzymes and continuous enzymatic reactions, among which glucose import, hexokinase, PFKFB3, lactate export, and other enzymes play essential roles in glycolysis (Eisenberg et al., 2020). These enzymes and processes may also be involved in OA pathogenesis. Figure 1 summarizes the glycolytic metabolic pathways of OA. Therefore, targeting glycolysis, particularly glucose transporters and regulatory enzymes involved in regulating OA metabolism, might lead to significant breakthroughs in OA therapy. Table 1 summarizes the critical targets of OA pathogenesis in glycolysis. Recent studies have shown that some natural products regulate glycolysis (Li et al., 2022b). Intervention in the glycolytic process is an accurate and feasible way to prevent and treat OA (Dong et al., 2022), and Table 2 lists natural products that could interfere with glycolysis. These natural products might be potential drugs to inhibit OA development.

**TABLE 1:** The key targets in the pathogenesis of OA in glycolysis.

Key target	Metabolic function	Role in OA pathogenesis
GLUT1	Transports glucose to the cell	Balances glucose levels, articular cartilage lacking the GLUT1 gene showed cytopenia and proteoglycan loss
HK2	Phosphorylates a hexose to a hexose phosphate	Overexpression of HK2 results in increased RNA expression levels of pro-inflammatory cytokines such as IL-6, IL-8, and MMPs in OA synovial tissue
PKM2	Dephosphorylates phosphoenolpyruvate to pyruvate	PKM2 knockdown inhibits OA chondrocyte proliferation, promotes apoptosis, and downregulates COL2α1 and SOX9 expression levels
PFKFB3	A key stimulator of glycolysis	Improves the cell viability of chondrocytes, reduces caspase 3 activation, and promotes the expression of aggrecan and collagen type II
LDHA	Converts pyruvate to lactate	Promotes ROS formation in chondrocytes in the inflammatory state, overexpression of LDHA disrupts metabolic homeostasis in synovial tissue

OA, osteoarthritis.

**TABLE 2:** Natural products that interfere with the glycolysis process.

Compound	Source	Molecular formula	Cas number	Role of glycolysis	Author, year
Dauricine	Vietnamese Sophora root	C_38_H_44_N_2_O_6_	524–17-4	↓HK2, PKM2	Li et al. (2018)
Monocrotaline	Crotalaria novae-hollandiae, Crotalaria recta	C_16_H_23_NO_6_	315–22-0	↓HK1	Zhang et al. (2014)
Oxymatrine	Sophora pachycarpa, Sophora chrysophylla	C_15_H_24_N_2_O_2_	16,837–52-8	↓PKM2, GULT1	Li et al., (2020c)
Sinomenine	Sinomenium acutum, Stephania cephalantha	C_19_H_23_NO_4_	115–53-7	↓HK2	Liu et al. (2020)
Apigenin	Verbascum lychnitis, Carex fraseriana	C_15_H_10_O_5_	520–36-5	↓PKM2	Zhao et al. (2021)
Cynaroside	Verbascum lychnitis, Carex fraseriana	C_21_H_20_O_11_	5,373–11-5	↓PKM2, PFKFB3, HK2	Pei et al. (2021)
Iridin	Iris milesii, Iris tectorum	C_24_H_26_O_13_	491–74-7	↓PKM2, Lactate, Glucose	(Ying et al., 2021; Xu et al., 2022)
Isoliquiritigenin	Pterocarpus indicus, Dracaena draco	C_15_H_12_O_4_	961–29-5	↓GLUT 1/4, HK2, PKM, LDHA	Wang et al. (2016)
Kaempferol	Lotus ucrainicus, Ardisia sanguinolenta	C_15_H_10_O_6_	520–18-3	↓PKM2, Lactate, Glucose	Wu et al. (2022)
Licochalcone A	Euphorbia helioscopia, Pogostemon cablin	C_21_H_22_O_4_	58,749–22-7	↓HK2	Wu et al., (2018b)
Quercetin	Plant food	C_15_H_10_O_7_	117–39-5	↓Glucose, Lactate, PKM2, GLUT, LDHA, HK2	(Jia et al., 2018; Wu et al., 2019)
Xanthohumol	Humulus lupulus L	C_21_H_22_O_5_	6,754–58-1	↓HK2	Yuan et al. (2020)
Catechin	Visnea mocanera, Salacia chinensis	C_15_H_14_O_6_	7,295–85-4	↓LDHA, Lactate	Han et al. (2021)
Epigallocatechin gallate	Limoniastrum guyonianum, Scurrula atropurpurea	C_22_H_18_O_11_	989–51-5	↓HK2	Gao et al. (2015)
Resveratrol	Grapes and other food products	C_14_H_12_O_3_	501–36-0	↓HK2, GLUT1, PFK1, PKM2	(Dai et al., 2015; Han et al., 2015; Li et al., 2016a; Wu et al., 2018a)
Rosmarinic acid	Dimetia scandens, Scrophularia scorodonia	C_18_H_16_O_8_	20,283–92-5	↓Lactate, Glucose	Han et al. (2015)
Astragaloside IV	Euphorbia glareosa, Astragalus ernestii	C_41_H_68_O_14_	84,687–43-4	↓LDHA	Zhang et al. (2018)
Cassiaside C	Senna obtusifolia, Senna tora	C_27_H_32_O_15_	119,170–52-4	↓Lactate	Kim et al. (2022)
Andrographolide	Andrographis paniculata	C_20_H_30_O_5_	5,508–58-7	↓PFKFB3	Yao et al. (2019)
Celastrol	Reissantia buchananii, Crossopetalum gaumeri	C_29_H_38_O_4_	34,157–83-0	↑ATP, Glucose, ↓LDHA, GLUT1, HK2, Lactate	Chen et al. (2022)
Costunolide	Magnolia garrettii, Critonia morifolia	C_15_H_20_O_2_	553–21-9	↓HK2, Lactate, Glucose	Ban et al. (2019)
Cryptotanshinone	Salvia miltiorrhiza, Salvia przewalskii	C_19_H_20_O_3_	35,825–57-1	↓GLUT1, LDHA, HK2, PKM2	(Yang et al., 2018b; Zhou et al., 2020)
Paclitaxel	Taxus brevifolia	C_47_H_51_NO_14_	33,069–62-4	↓GLUT1, PKM2, LDHA	Long et al. (2020)
Shikonin	*Echium plantagineum*, Arnebia hispidissima	C_16_H_16_O_5_	517–89-5	↓GLUT1, PKM2, HK2	(Vališ et al., 2016; Zhao et al., 2018; Li et al., 2021; Zhang et al., 2021; Dai et al., 2022; Huang et al., 2022)
Tanshinone IIA	Salvia miltiorrhiza, Salvia digitaloides	C_19_H_18_O_3_	568–72-9	↓HK2	Li et al., (2020b)
Atractylodin	Atractylodes japonica, Atractylodes macrocephala	C_13_H_10_O	55,290–63-6	↓Lactate	Qu et al. (2021)
Cinnamic acid	Plantago coronopus, Marsypopetalum crassum	C_9_H_8_O_2_	140–10-3	↓PKM2	Yao et al. (2021)

↑, increase; ↓, decrease.

## GLUT1

Glucose is the main energy substrate for chondrocytes and the main precursor for glycosaminoglycan synthesis. When glucose levels rise, chondrocytes, if unable to adjust, absorb more glucose and produce more reactive oxygen species. Increased extracellular glucose levels also lead to increased production of AGEs, leading to cartilage damage. Glucose import is the first rate-limiting step in chondrocyte glycolysis (Mobasheri et al., 2002). Glucose is transported into chondrocytes by GLUT1, which is upregulated in hypoxia and glucose deprivation and decreased in high-glucose environments. GLUT1 improves the ability to take up glucose under hypoxic conditions and balances glucose levels in cells (Peansukmanee et al., 2009). Articular cartilage lacking the GLUT1 gene shows cytopenia and proteoglycan loss, which can seriously aggravate OA injury. Consistently elevated GLUT1 expression degrades cartilage by increasing glucose uptake and producing excessive AGEs (Rasheed et al., 2011). It might play a potential role in OA pathogenesis.

## HK2

HK2 is the first rate-limiting enzyme in glycolysis, which can catalyze the conversion of glucose to glucose-6-phosphate (G-6-P) and participate in the main glycolysis pathway (Bao et al., 2022). A subtype of HKs, HK2 is a key regulator of glucose metabolism, promoting the conversion of glucose metabolism from oxidative phosphorylation to aerobic glycolysis (Feng et al., 2020). The expression level of HK2 in OA synovial tissue (FLS) is higher than that in the normal group. Overexpression of HK2 results in increased RNA expression levels of pro-inflammatory cytokines, including IL-6, IL-8, and MMPs in OA FLS. This suggests that HK2 has a potential therapeutic effect on OA (Bustamante et al., 2018). Identifying metabolic targets in the development of OA therapy is of great significance.

## PKM2

PKM2 is a rate-limiting enzyme in glycolysis. It has been reported that PKM2 is upregulated, and ATP production is decreased in OA chondrocytes. Inhibition of PKM2 can prevent the proliferation of OA chondrocytes, promote cell apoptosis, and reduce the expression levels of COL2α1 and SOX9. When PKM2 is overexpressed in OA chondrocytes, it leads to lactate accumulation and the formation of an acidic microenvironment (Yang et al., 2018a). The acidic microenvironment inhibits matrix synthesis in chondrocytes and may promote cartilage degeneration in OA (High et al., 2019), suggesting PKM2 might be a therapeutic target for OA (Yang et al., 2018a).

## PFKFB3

PFKFB3 is a crucial stimulator of glycolysis. PFKFB3 is down-regulated in human OA cartilage tissues, and human chondrocytes are stimulated with TNF-α or IL-1β. PFKFB3 overexpression ameliorates the damaged glycolytic process in OA cartilage. In addition, PFKFB3 can improve the cell viability of chondrocytes, reduce the activation of caspase-3, and promote the expression of aggrecan and type II collagen, which is a potential target for OA prevention and treatment (Qu et al., 2016).

## LDHA

LDHA is closely related to aerobic glycolysis. It is essential for lactate production (Errea et al., 2016; Kaushik et al., 2019). Overexpression of LDHA disrupts metabolic homeostasis in synovial tissue, which directly upregulates lactate secretion. Extracellular lactate can stimulate histone lactation. Hypoxia enhances glycolysis, increases intracellular lactate levels, and increases histone lysine lactylation (Kla) levels (Pan et al., 2022). It has been found that lactate reduces M1 macrophage activation and promotes M2 macrophage polarization by modifying histones in the inflammatory response (Zhang et al., 2019; Ivashkiv, 2020). Further studies on histone lactation may lead to new therapeutic targets for OA treatment and strategies for resolving inflammation (Zhou et al., 2022). The activity and expression of LDH are significantly increased in primary chondrocytes treated with IL-1β. The mRNA sequencing results showed that IL-1β induction significantly increased the expression of genes involved in glycolysis, including PKM2, LDHA, and HK2 (Arra et al., 2020). In addition, the levels of LDHA and lactate in synovial fluid of TMJOA patients are significantly higher than those of controls (Li et al., 2020a). LDHA can promote ROS formation in chondrocytes in an inflammatory state. Inhibition of LDHA activity is an effective therapeutic target for OA.

## Conclusion

In conclusion, improving the metabolic environment is conducive to treating OA, primarily by regulating glycolysis. Articular cartilage lacking GLUT1 exhibits cytopenia and proteoglycan loss. Inhibition of PKM2 could prevent the proliferation of OA chondrocytes and reduce the expression levels of COL2α1 and SOX9. PFKFB3 can improve the cell viability of chondrocytes and promote the expression of aggrecan and collagen type II. LDHA is closely related to glycolysis and plays a vital role in chondrocyte proliferation and apoptosis. Lactate, a byproduct of glycolysis, is a vital signal determining inflammation. Lactate can inhibit inflammatory responses by blocking pro-inflammatory signaling pathways. In addition, lactate exerts anti-inflammatory effects by promoting the polarization of M2 macrophages after modification of histone lactylation. Further studies of lactate may identify new therapeutic targets for treating OA and resolving inflammation.

Many researchers are interested in the role of glycolysis in OA pathogenesis. However, comprehensive details of its specific regulatory mechanisms and signaling pathways are yet to be studied. In addition, many natural products have been shown to regulate glycolysis, but there are few reports that natural products inhibit OA *via* glycolysis, which could provide a new strategy for OA treatment. Targeting glucose transporter proteins and rate-limiting enzymes in glycolysis may have a tremendous breakthrough in OA treatment. By discussing glycolysis and OA, we believe that targeting glycolysis is an important strategy to treat OA. And natural products have great advantages for improving OA.
